# Macrophage Involvement in Medication-Related Osteonecrosis of the Jaw (MRONJ): A Comprehensive, Short Review

**DOI:** 10.3390/cancers14020330

**Published:** 2022-01-10

**Authors:** Ioannis Gkouveris, Akrivoula Soundia, Panagiotis Gouveris, Dionysia Zouki, Danny Hadaya, Sotirios Tetradis

**Affiliations:** 1 Division of Diagnostic and Surgical Sciences, UCLA School of Dentistry, Los Angeles, CA 90095, USA; gkouver@dent.uoa.gr (I.G.); akrivi@ucla.edu (A.S.); dhadaya@ucla.edu (D.H.); 2 Second Department of Medical Oncology, Agios Savvas Cancer Hospital, 11522 Athens, Greece; pgouver@yahoo.com (P.G.); dzouki@med.uoa.gr (D.Z.)

**Keywords:** antiresorptives, macrophages, osteonecrosis, inflammation

## Abstract

**Simple Summary:**

Medication-related Osteonecrosis of the Jaw (MRONJ) is a significant complication mainly of antiresorptive medications used in the management of bone diseases. MRONJ development may be accompanied by pain, eating discomfort, self-consciousness, and other symptoms that overall disturb patients’ everyday life. Hence, MRONJ occurrence is of growing clinical concern and affects treatment decisions. Although MRONJ has been extensively studied since being first reported in 2003, the mechanisms of disease pathogenesis have not yet been determined and disease management is mostly empirical. Recent data investigate the effects of antiresorptive medications on immune system components including macrophages and introduce these cells as key players in MRONJ pathogenesis. Considering macrophage versatility, developmental plasticity, and its pivotal role in immune response, the current short review focused on the potential involvement of these multi-potential cells in MRONJ pathogenesis. Understanding the complex role of macrophages in MRONJ pathophysiology will add new valuable data on disease prevention and control.

**Abstract:**

Antiresorptive agents such as bisphosphonates (BP) and denosumab are commonly prescribed for the management of primary bone malignancy, bone metastasis, osteoporosis, Paget disease, or other bone disorders. Medication-related osteonecrosis of the Jaws (MRONJ) is a rare but significant complication of antiresorptive medications. Duration, dose, and antiresorptive potency as well as concomitant diseases, additional medications, and local factors affect MRONJ incidence and severity. MRONJ pathophysiology is still poorly understood. Nevertheless, decreased bone resorption due to osteoclastic inhibition along with trauma, infection/inflammation, or blood supply inhibition are considered synergistic factors for disease development. In addition, previous data research examined the effects of antiresorptive medication on immune system components and introduced potential alterations on immune response as novel elements in MRONJ pathogenesis. Considering that macrophages are the first cells in the nonspecific immune response, it is not surprising that these multifaceted players attracted increased attention in MRONJ research recently. This current review attempted to elucidate the effects of antiresorptive medications on several aspects of macrophage activity in relation to the complex inflammatory microenvironment of MRONJ. Collectively, unravelling the mode of action and extent of macrophages’ potential contribution in MRONJ occurrence will provide novel insight in disease pathogenesis and potentially identify intrinsic therapeutic targets.

## 1. Introduction

Medication-related osteonecrosis of the jaws (MRONJ) is a rare but potentially severe adverse effect of antiresorptive and, more recently, antiangiogenic medications administered to patients with osteoporosis or bone malignancies [1,2]. MRONJ presents as exposed bone in the maxillofacial region or bone that can be probed through an intraoral or extraoral fistula, which does not heal for 8 weeks, in patients under antiresorptive/antiangiogenic medication without previous exposure in radiotherapy in the head and neck area [1]. Common antiresorptive agents largely involved in MRONJ are bisphosphonates and denosumab, a humanized monoclonal antibody against Receptor Activator of Nuclear Factor κΒ Ligand (RANKL).

MRONJ can pose challenges for its clinical management. Most of the treatment interventions are empirical and do not address the pathophysiology of the disease [3,4]. Conservative and surgical interventions have been utilized both with benefits and drawbacks [3,4,5,6]. Surgical treatments appear to be effective; however, they can often result in large defects that compromise function [3,7]. Furthermore, patients with MRONJ can present with concomitant medical conditions that limit treatment options with high morbidity. Experimental therapeutic strategies have utilized mediators that promote healing, such as platelet-rich plasma (PRP), platelet-rich fibrin (PRF), plasma rich in growth factors (PRGF), ozone oil, or Vitamin E as well as hyperbaric oxygen with variable results [4,5,8,9].

Even though MRONJ was initially described in 2003, its pathophysiology has not been fully elucidated yet [10,11]. Decreased bone resorption due to osteoclastic inhibition along with trauma, infection/inflammation, or blood supply suppression have already been implicated as synergistic factors for disease development [11]. However, the impact of each factor is still arguable, while none is adequate to explain the full spectrum of MRONJ presentation [1,10,11,12]. Antiresorptive medications in conjunction with local instigating factors, such as gingival/periodontal disease or tooth extraction, increase MRONJ risk [11]. Interestingly, although tooth extraction is reported as the most common local instigating factor for MRONJ development [1], recently it was reported that after adjustment for confounders, tooth extraction does not increase MRONJ risk, suggesting that extraction of infected teeth in patients on antiresorptives should be performed to eliminate local infection [7,13].

More recent data investigate the effects of antiresorptive medications on immune system components and introduce potential alterations on immune response as novel elements in MRONJ pathogenesis [14,15,16]. Indeed, several studies suggest antiresorptive-related alterations on important cells of both innate and immune inflammation, including T-cells, dendritic cells (DCs), polymorphonuclear neutrophils, and macrophages [14,17,18,19,20,21]. Peripheral blood of mice with MRONJ-like lesions showed lower regulatory T-cell (Treg) numbers and significant prevalence of Th17 cells and IL-17 levels, potentially contributing to the prolonged inflammation at sites of the disease [20]. Likewise, a significant increase in Th17 cells and IL-17 expression was reported at mucosal tissues adjacent to non-healing extraction sockets of MRONJ patients [22]. Significantly higher γδ-T cell levels were seen in patients with MRONJ lesions compared to healthy individuals, while γδ-T cells co-cultured with osteoclasts secreted large amounts of IFN-γ following zoledronic acid (ZA) treatment [23]. Bisphosphonates (BPs) inhibited DC maturation and activation and impair phagocytosis, potentially promoting immunosuppression or infectious complications [24]. ZA administration impaired DC functions and increased bacterial load in the oral cavity of mice, while DC-deficient mice presented higher osteonecrosis rates following dental extraction [18]. BPs also affected neutrophil activity and induced pro-inflammatory effects [19]. In vitro experiments suggested that BPs shorten neutrophil life span without affecting their differentiation [21]. In vivo animal studies indicated that BPs increase neutrophil numbers as well as related pro-inflammatory cytokines, including Tumor Necrosis Factor (TNF)-α, IL-1β, inducible nitric oxide synthase (iNOS), NF-kβ, and IL-18 binding protein at MRONJ sites [17]. In addition, in vitro studies indicated that BPs affect multiple cell types including endothelial cells, periodontal fibroblasts, periodontal stem cells, and osteoblasts [25,26,27,28,29].

Cells of the monocyte/macrophage lineage are key players in inflammation, from the early phases of acute response to the late stages of resolution [30,31,32]. Macrophages are multifunctional cells involved in complex processes that can exacerbate tissue damage or promote wound healing and, thus, have been investigated in the setting of MRONJ [31,33,34,35]. Here, we focused our review on the existing evidence of the potential contribution of macrophages in MRONJ pathophysiology.

## 2. Macrophage Involvement in MRONJ Pathophysiology

### 2.1. Macrophage Biology

Macrophages comprise a heterogeneous population of myeloid cells, involved in critical innate immune system responses, present tissue-specific activity, and serve as key regulators of organ health and homeostasis [32]. Tissue-localized macrophages demonstrate crucial functions including pathogen recognition and modulation of adaptive immune responses, as well as tissue healing and regeneration [32,36]. Macrophages are important players in the initiation, maintenance, and resolution of inflammation and are activated and deactivated during progression of the inflammatory process [30]. They express and secrete several cytokines, extracellular matrix proteins, and chemical mediators that either serve as pro-inflammatory signals or deactivate mediators and induce proliferation and remodeling, thus favoring tissue repair and wound healing [31].

Macrophages have great plasticity and adapt distinct functional phenotypes, a process known as macrophage polarization [37,38,39]. Typically, macrophages polarize into classically activated (M1) and alternatively activated (M2) phenotypes [39]. Classically activated pro-inflammatory M1 polarization occurs in response to lipopolysaccharide (LPS) and Th1-released cytokines (such as IFNγ and TNFa). In turn, M1-polarized macrophages express Toll-like receptors (TLR) 2, TLR-4, CD80, CD86, iNOS, and MHC-II surface proteins and secrete cytokines and chemokines such as TNFa, IL-1a, IL-1b, IL-6, IL-12, CXCL9, and CXCL10 that further promote M1 polarization of existing unpolarized macrophages, thus providing a constant positive feedback loop [40,41,42]. M1 macrophages drive tissue damage with microbial and anti-tumor activity [43]. Conversely, M2 polarization arises following stimulation by IL-4, IL-13, IL-10, and IL-33 cytokines and TGF-b [44]. M2 macrophages are further divided into M2a, M2b, M2c, and M2d subcategories depending on their expression of specific surface markers including CD206, CD209, CD163, mannitol receptor, and Ym1/2 [39]. M2 macrophages support an anti-inflammatory environment by promoting angiogenesis and tissue regeneration; however, the same cells may favor tumor formation and progression in a malignant microenvironment [43].

Monocytes, macrophages, DCs, and osteoclasts belong to the family of mononuclear phagocytes and present common but distinct properties [45]. Even though monocytes, macrophages, and DCs derive from a common monocyte-macrophage DC progenitor (MDP) [46], in adult healthy tissues, resident macrophages either derive from circulating monocytes or from embryonic precursors that seed the tissues before birth [47,48]. However, accumulating macrophages at diseased sites typically arise from circulating monocytes [49]. Osteoclasts arise from bone marrow precursors in healthy conditions, while osteoclasts may evolve from mononuclear cells and DCs in pathological states [50,51].

Macrophages’ versatility and developmental plasticity and their pivotal role in the immune response can explain the potential involvement of these multi-potential cells in MRONJ pathogenesis. Several lines of evidence introduce macrophages as prospective players in MRONJ pathophysiology. Published research examines the effect of antiresorptive medication on macrophage growth, as well as other macrophage functional processes including survival, apoptosis, or migration. Other studies seek modifications in macrophage polarization status followed by relative alterations in cytokine expression. Both in vitro and in vivo experimental approaches have been employed, each with distinct advantages and disadvantages. Nevertheless, both experimental approaches point to a significant role of macrophages in MRONJ pathogenesis.

### 2.2. In Vitro Studies

**Figure 1:**
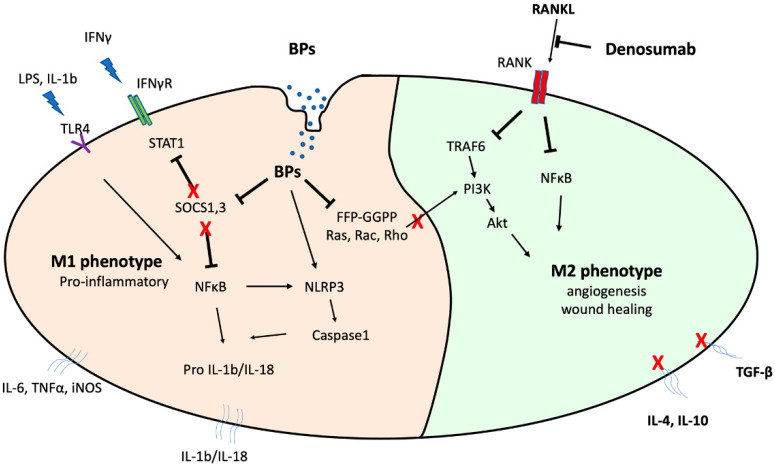
In vitro effects of antiresorptives on macrophage signaling cascades. BPs’ internalization by macrophages results in inhibition of the mevalonate pathway and SOCS1/3 signaling and augments NLRP3 inflammasome activity. Moreover, BPs enhance IFNγ/STAT1 and TLR4 signaling, driving to NFκB hyper-activity. In addition, downregulation of key mevalonate enzymes inhibits PI3K-dependent M2 polarization. Altogether, BPs favor M1 polarization and contribute to a pro-inflammatory phenotype that results in mature IL1b/IL18 synthesis as well as IL6, iNOS, and TNFα release. Denosumab administration inhibits RANK downstream signaling pathways, including NFκΒ- and PI3K/AKT-dependent M2 polarization. (NLRP3, NOD-like receptor proteins 3; SOCS, Suppressors of cytokine signaling; STAT, Signal transducer and activator of transcription; PI3K, Phosphoinositide 3 kinases).

A list of key references with important in vitro along with a few in vivo (mice) findings of antiresorptives on macrophage function are seen in Table 1, while a summary of the potential in vitro antiresorptive effects on macrophage activity is presented in Figure 1.

### 2.3. Alterations in Macrophage Growth and Function

Macrophages are crucial players in inflammation and present phagocytic activity; therefore, it is likely that, along with their blood progenitors, they may be affected by BPs through direct drug internalization [52]. During the past decade, several studies attempted to investigate the direct effect of BPs on macrophage activity, related to MRONJ occurrence.

Furthermore, few researchers have examined BP effects on immortalized human THP-1 monocytic cells [53]. Phorbol-12-myrisate-13-acetate (PMA) enhances THP-1 differentiation to macrophages followed by increased CD68 expression and high cell-adherence efficacy [33]. In contrast to denosumab, Zoledronic acid (ZA) addition results in significant macrophage detachment and reduced cell viability in a dose-dependent manner [33]. Similarly, six commonly prescribed bisphosphonates (zoledronate, ibandronate, risedronate, alendronate, pamidronate, and clodronate) hamper THP-1 cell adherence and survival in a dose- and time-dependent manner [53]. Moreover, both ZA and alendronate reduce THP-1-derived macrophage survival, cause morphological changes, and impair both their mRNA and activity levels [52]. In addition, high ZA concentrations affect PMA-induced THP-1 differentiation, as observed by the reduced CD68 expression levels, while alendronate only triggers such an effect at higher concentrations [52]. Likewise, Hoefert et al. proposed that high doses of zoledronate, ibandronate, or alendronate reduced time to maximum migration of THP-1 cells, disturbed phagocytosis, and resulted in alterations of the actin cytoskeleton [54].

In concert with other players, two recent studies examined the effects of ZA administration on murine macrophage cell line, RAW264.7. ZA-treated macrophages present high apoptotic rates, which is further induced by LPS stimulation [57]. Both free and calcium-bound BPs significantly reduce RAW264.7 macrophage cell viability and absolute cell numbers in vitro [56].

The direct effect of anti-RANKL mAb on cell cultures of mice Bone Marrow-Derived Macrophages (BMDM) and osteoclasts as well as on mice gingival fibroblasts was investigated [66]. In vitro experiments demonstrated that mAb suppressed osteoclast numbers but did not affect fibroblast and macrophage populations [66]. Similarly, denosumab administration to THP1 cells or RANKL-ab treatment of mouse BMDM did not trigger any alteration in macrophage numbers or viability [33,67].

### 2.4. BP Mechanism of Action against Macrophages

On the basis of these numerous reports linking BP administration with various aspects of macrophage biology, it is reasonable to speculate that BPs directly impair monocyte to macrophage differentiation, reduce their survival rate, and induce morphological alterations that overall disrupt their activities [52]. As discussed above, not only osteoclasts but also macrophages and monocytes can be affected by BP administration [52,71], and the extent of this effect depends on their endocytic activity, uptake as well as the type of administered BP [52,53,71]. For instance, ZA demonstrates a higher inhibitory effect on cell viability [53] and differentiation than ALN [52].

An additional mechanism of action of the more potent nitrogen-containing bisphosphonates ((NBPs) zoledronate, risedronate, ibandronate, alendronate) is the inhibition of key enzymes of the mevalonate pathway, including farnesyl pyrophosphate synthase (FPPS), which produces FPP. In turn, FPP contributes to the generation of geranylgeranyl pyrophosphate (GGPP) [72]. FPPS inhibition impairs biosynthesis of isoprenoid compounds essential for the post-translational prenylation of crucial small GTPases [21,57,73]. Therefore, NBPs may induce apoptosis by inhibiting the mevalonate pathway [74], since small GTPases such as Rab, Rho, and Rac are key modulators of cell proliferation, apoptosis, and migration in several cells including osteoclasts [21]. In particular, Rac1 and Rho are involved in types 1 and 2 internalization pathways, respectively, whereas phagocytic efficacy is also related to macrophage mannose receptor (CD206) and CD68 scavenger receptor [75]. Similarly, Rac contributes to Fcγ-receptor-mediated phagocytosis [76] as well as to migration by transducing cell surface signaling to actin and microtubules [76,77].

In an effort to explore manipulation of geranylation in support of osteoclast function, Kimachi et al. determined that ZA treatment downregulated RANK expression and inhibited migration of RAW264.7 monocyte/macrophage precursor cells [58]. Geranylgeraniol (GGOH) addition partially reversed this ZA-dependent inhibitory effect [58]. Similarly, farnesyl-pyrophosphate and geranylgeranyl pyrophosphate treatment of THP-1 cells completely rescued mevalonate pathway inhibition and migration reduction, whereas migration was initially inhibited after simvastatin administration [78]. Furthermore, macrophages derived from geranylgeranyl transferase-I deficient mice are hyper-activated by LPS, while protein geranylgeranylation favors K-Ras GTPase crosstalk with the PI3K catalytic subunit p110δ [79]. Of note, geranylgeranylation inhibition impairs PI3K activation and results in excessive IL-1β secretion by macrophages and constant activation of pyrin inflammasome [79]. Likewise, mevalonate pathway inhibition in human PBMC cells downregulates small GTPases’ (Rho, Rac, Rap) expression followed by inflammasome-mediated caspase-1 activation and IL-1ß release [80].

Moreover, ZA-treated RAW264.7 macrophages present increased caspase-1 and IL-1β mRNA expression and decreased histone H3k27me3 protein levels [60]. Similarly, ZA administration significantly increased the mRNA and protein levels of Kdm6a and Kdm6b proteins; the latter specifically mediate demethylation of H3k27me3/2 peptides [60]. In contrast, Kdm6a and Kdm6b knockdown suppressed caspase-1 and IL-1β protein expression in ZA-stimulated RAW264.7 macrophages.

### 2.5. Alterations in Macrophage Protein Expression

In addition to effects on macrophage function and growth, BPs alter macrophage protein expression. In general, BPs either alone or in combination with other signals enhance pro-inflammatory cytokine production and release, and, thus, have been hypothesized to augment and sustain an inflammatory environment.

ZA administration enhances leptin-induced IL-6 expression in mouse BMDM and downregulates SOCS3 expression in human BMDM [63]. ZA treatment of mouse BMDM reduces STAT3 phosphorylation, while farnesol addition restores the pSTAT3 inhibitory effect, suggesting that ZA modulates STAT3 signaling through a mevalonate-dependent pathway. Another interesting observation of this study indicated that SOCS3 expression is significantly decreased in human MRONJ tissues compared to healthy controls [63].

Moreover, in an effort to explore potential effects of ZA on LPS-induced cytokine expression by macrophages, Muratsu et al. pretreated murine RAW264.7 cells with ZA for 24 h and then added LPS [57]. ZA administration enhanced LPS-triggered expression of IL-1b, IL-6, TNF-a, and NO. Specifically, ZA treatment attenuated SOCS1 expression, resulting in upregulation of NFkB signaling and excessive cytokine production. Nevertheless, ZA administration alone did not result in any significant cytokine release from RAW264.7 cells [57]. Similarly, alendronate directly enhances IL1 expression by RAW264.7 cells, while clodronate, a non NBP, inhibited this activity [59]. In turn, the authors intraperitoneally injected alendronate in mice and suggested that IL1 expressed in tissues was mainly in a pro-IL-1 status, since alendronate could slightly stimulate caspase-1 (pro-IL1 activator), whereas pro-IL1 increase was hardly noticeable in macrophage-depleted mice [59].

In addition, Morita et al. [61] cultured mouse BMDM with M-CSF and RANKL and concluded that in the presence of alendronate BMDM overexpressed TNFα, IL-6, and IL-1β. Moreover, they proposed that alendronate treatment may promote osteonecrosis development in mice femur with existing osteomyelitis while TNFα gene or chemical inhibition prevents MRONJ incidence. Additionally, IL-6 or IL-1α/β deficient mice presented considerably reduced osteonecrosis events [61]. In accordance, elevated IL6 and reduced IL10 expression levels correlated with advanced MRONJ stages in an immunohistochemical study of both denosumab and BP-treated patients presenting stage I–III MRONJ [35].

BPs appear to not only impair macrophage differentiation and survival but also to modulate their functions at mRNA activity levels [52]. Indeed, ZA administration in THP-1-derived macrophages results in upregulation of MMP2 and MMP9 mRNA levels. Therefore, it was speculated that excessive MMP-mediated tissue degradation at areas of bone injury may delay wound healing, thus driving to bone necrosis and subsequent MRONJ development [52].

On the other hand, it is still under investigation whether denosumab modulates cytokine expression by macrophages. Recently, it was reported that RANKL-Ab treatment significantly decreases the number of osteoclasts and suppresses osteoclast markers but does not affect the relative expression levels of IL-10 and TGF-β of mice BMDM [67]. It was also already mentioned that denosumab administration does not seem to interfere with monocyte to macrophage differentiation or macrophage viability [33,68,69]. Nonetheless, osteoclasts derived from human PBMC secrete a profile of chemokines and cytokines that closely resemble those of M2 macrophage phenotype [81]. Therefore, denosumab treatment by inhibiting osteoclast differentiation and function could indirectly downregulate M2-secreted proteins without necessarily exerting a direct effect on macrophage function.

In summary, there is accumulating in vitro evidence to suggest that BPs and in particular NBPs might disturb local immune function of macrophages in bisphosphonate-related ONJ (BRONJ) by directly affecting their survival, migration, differentiation, and phagocytic activity, thus introducing macrophages as new key players in BRONJ pathogenesis. On the other hand, macrophage studies on denosumab-related ONJ (DRONJ) are critically few and the limited data remain controversial; nevertheless, the majority of evidence suggests no direct effect of denosumab on macrophage activity.

### 2.6. Alterations in Macrophage Polarization

Over the past decade, several studies examined the role of macrophages in MRONJ pathophysiology and suggested that alterations in macrophage polarization may contribute to the sustained inflammatory microenvironment and delayed tissue healing in MROJN sites. However, the process of antiresorptive interference on macrophage polarization and related MRONJ pathogenesis has not been fully elucidated.

Interestingly, Kaneko et al. (2018) differentiated THP-1 cells to macrophages by applying PMA and then added ZA in the cell cultures for an additional 24 h. In turn, they administered LPS and IL4 and tested effects on M1 and M2 polarization, respectively [55]. ZA treatment induced LPS-mediated M1 polarization but did not affect M2 macrophage rates [55]. The authors suggested that ZA induces LPS-mediated M1 polarization through an NLRP3 inflammasome signaling pathway [55]. These findings are consistent with previous in vitro studies describing that NLRP3 expression is induced by LPS in M1 but not in M2 macrophages [82]. In contrast, NLRP3 inflammasome inhibition results in reduced M1 polarization and augments M2 phenotype [83]. Others previously investigated the correlation between TLR-4 expression and macrophage polarization as well as subsequent MRONJ development [16]. TLR-4 was the predominant TLR expressed in macrophages after ZA treatment of mouse bone marrow-derived macrophages (BMDM). ZA treatment of BMDMs upregulates CD86-expressing M1 and downregulates CD206-expressing M2 macrophages. Moreover, TLR-4 signaling inhibition downregulates the TLR-4/NF-kB downstream pathway and suppresses ZA-enhanced pro-inflammatory cytokine secretion by macrophages and, thereby, M1 phenotype prevalence [16].

## 3. Clinical Observations and In Vivo MRONJ Animal Models

The above in vitro studies suggest that BPs and in particular NBPs disturb macrophage growth, differentiation, and function, thus pointing to macrophages as potential key regulators of MRONJ pathogenesis. On the other hand, in vitro studies are limited in capturing the full spectrum of the far more complex in vivo conditions. Although few studies on patients or on MRONJ animal models have been conducted, they provide valuable insights on the involvement of macrophages in the pathogenesis of the disease.

### 3.1. Macrophages May Modulate MRONJ Incidence and Wound Healing

Macrophages around osteonecrotic lesions in BP-treated mice demonstrate a heightened inflammatory response. Elevated IL6 and reduced IL10 expression levels are present in tissues of patients treated with BPs or denosumab and correlate with advanced stages of MRONJ, thus pointing to an enhanced pro-inflammatory environment [35]. CD14 is seen in monocyte/macrophage lineage cells, while CD68 is expressed in mature macrophages and is connected to increased phagocytic activity. A lower CD68/CD14 rate is noted in BRONJ lesions compared to lesions of osteoradionecrosis or secondary chronic osteomyelitis, pointing to a macrophage immunosuppression in BRONJ tissues. Importantly, this immunosuppression does not depend solely on the effect of BP, since healthy tissues of patients on BPs do not present an altered CD68/CD14 ratio [34].

Epigenetic alterations regulate inflammatory gene transcription, while histone methylation correlates with both gene repression and activation of transcription [60]. GSK J4, a selective inhibitor of H3K27 histone, rescues poor healing of alveolar sockets and decreases numbers of CD11b^+^ macrophages and caspase-1 and IL-1β expression in ZA-treated mice [60]. BMDM from diabetic vs. healthy mice secrete higher IL-1β levels following treatment with NLRP3 inflammasome inducers, and ZA enhances this effect [62]. Macrophages overexpressing NLRP3, caspase-1, and IL-1β correlate with delayed extraction socket healing and bone necrosis in diabetic vs. control mice treated with ZA [62]. Intraperitoneal administration of either Ac-YVAD-cmk, (a caspase-1 inhibitor) or glyburide (an NLRP3 inhibitor) impairs NLRP3 activity and ameliorates BRONJ incidence in diabetic mice followed by a relevant decrease in Il-1β serum levels [62]. Additionally, MRONJ incidence and M1 macrophage numbers are attenuated in TLR4-/- mice, and TAK-242, a TLR-4 inhibitor, lowers MRONJ rate and improves wound healing in non-genetically modified mice [16].

The above findings suggest that BP treatment either directly or indirectly creates an environment of heightened and prolonged inflammatory response, increases pro-inflammatory cytokine release, and alters macrophage polarization that collectively propagates tissue damage and attenuates wound healing.

Most in vivo animal studies have utilized BPs to induce MRONJ-like lesions, with only a few studies exploring the effect of RANKL inhibitors on the macrophage involvement in MRONJ pathogenesis. From transgenic studies, it has been reported that RANKL expression is not essential for monocyte/macrophage differentiation and functional maturation in RANKL^−/−^ mice at baseline [68,69]. In contrast, in vivo inhibition of RANKL activity during inflammation-mediated arthritis reduces monocyte/macrophage function and ameliorates arthritis incidence in mice [70]. Interestingly, subcutaneous anti-RANKL mAb administration following palatal bone denudation surgery in mice results in delayed wound healing and increases adjacent tissue inflammation [66]. These findings indicate that macrophages might be involved in the altered bone healing in the presence of RANKL inhibitors. Whether this macrophage involvement is a direct effect of the inhibitors on macrophages or whether this is due to inhibition of bone resorption remains unclear.

### 3.2. Macrophage Polarization Changes in MRONJ Tissues

Mucosal tissues adjacent to osteonecrotic areas of patients with stages I–III MRONJ demonstrate a significant increase in M1 density and M1/M2 ratio, while the M2 population is decreased in advanced disease stages. In addition, M1 and M2 density is significantly higher in patients under bisphosphonates vs. denosumab treatment. Thus, a high M1/M2 ratio correlates with advanced MRONJ [35]. Similarly, BRONJ specimens display a significantly higher M1 infiltration compared to controls, thus indicating that an M1 polarization shift may be associated with BRONJ pathogenesis [65].

Additionally, IL-17 expression and increased M1 and decreased M2 macrophage infiltration is seen in established BRONJ sites in cancer patients and in jaw lesions of a BRONJ-like mouse model. IL-17 induces IFN-γ-mediated M1 polarization by enhancing STAT1 phosphorylation, while reducing IL-4-mediated M2 differentiation by inhibiting STAT6 signaling in vitro. Importantly, in mice, an IL-17 neutralizing antibody or Laquinimod (an IL-17 chemical inhibitor) reduces M1/M2 ratio and ameliorates BRONJ incidence [22].

In a mouse MRONJ model, animals were treated with cyclophosphamide (CY) and anti-mouse RANKL monoclonal antibody (mAb) or zoledronate combination treatment (CY/mAb and CY/ZA) before maxillary first molar extractions. Expression of F4/80, a macrophage marker and lymphatic vessel endothelial hyaluronan receptor 1 (LYVE-1), which is also expressed in macrophages, was investigated in MRONJ tissues. CY/ZA or ZA alone significantly reduced macrophage numbers in soft tissues adjacent to extraction sockets. In contrast, mAb and CY monotherapy affected neither F4/80^+^LYVE-1^−^ macrophages nor F4/80^+^LYVE-1^+^ cells. It was also noted that decreases in angiogenesis and lymphangiogenesis in areas of impaired healing were followed by predominance of F4/80^+^LYVE-1^−^ macrophages’ population [64]. Thus, the two antiresorptives, in combination with chemotherapy, might mediate their adverse effects through distinct mechanisms in this animal model of MRONJ. However, few studies explore the effects of RANKL antibodies or denosumab on angiogenesis. In mice treated with either ZA or OPG-Fc (denosumab analog) for 4 weeks, reduced arteriole and venule networks as well as decreased VEGF-A and VCAM1 expression levels at areas of experimentally induced MRONJ were observed [12].

Summarized in vivo (mouse) findings of antiresorptives on macrophage function in MRONJ pathophysiology are presented in Table 1.

## 4. Conclusions

Given their central role in nonspecific, innate immune response and multipotential function in wound healing, it is not surprising that macrophages have attracted increased attention in MRONJ pathogenesis research. In vitro studies suggest that BPs and in particular NBPs disturb local immune function of macrophages by directly affecting their survival, migration, differentiation, and phagocytic activity. In contrast, no direct effect of denosumab on macrophage has been described. However, in vitro studies on a cell culture level are limited in capturing the full spectrum of the far more complex in vivo conditions. On the other hand, clinical or in vivo translational studies clearly capture an altered macrophage function in BP or RANKL inhibitor-associated MRONJ.

MRONJ associated with either BPs or denosumab is clinically, radiographically, and histologically identical, despite the distinct molecular mechanism of action, pharmacokinetics, and clearance of these medications. Given that the primary action of both pharmacologic agents is osteoclast inhibition, a direct effect on macrophages in MRONJ pathogenesis remains uncertain. Furthermore, a direct effect of BPs should hinder macrophages at all skeletal sites. Ss such, the nearly exclusive occurrence of skeletal side effects to the jaws with MRONJ or to the femurs with atypical femoral fractures (AFFs) is puzzling [84].

**Figure 2:**
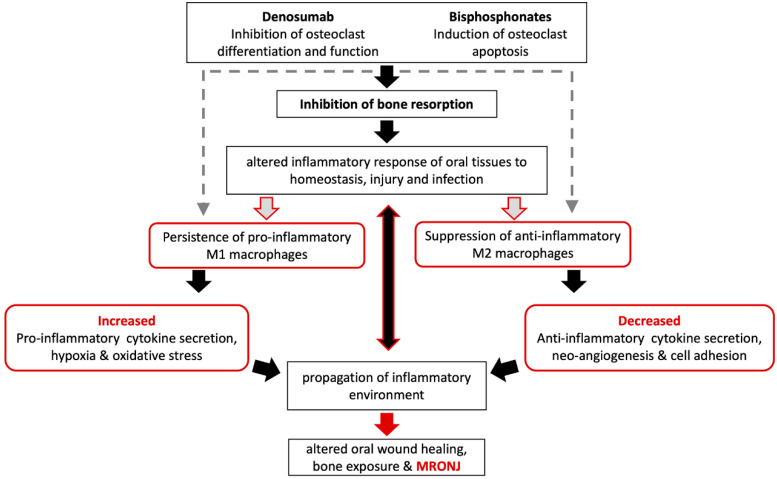
In vivo potential antiresorptives’ effect on macrophages’ Inhibition of bone resorption is central in Medication-Related Osteonecrosis of the Jaw (MRONJ) pathogenesis. Changes in macrophage polarization and function are likely in response to a sustained inflammatory environment and propagate the MRONJ extent and severity. Red color were used to highlight pathways and processes related to macrophahe biology that potentially contribute to MRONJ development.

Based on the in vitro and in vivo findings described above, a model of macrophage involvement in MRONJ pathophysiology was proposed (Figure 2). Any direct effect of antiresorptives on macrophages in the MRONJ process is speculative (broken, gray arrows).

Decreased osteoclast function and subsequent inhibition of resorption remain central in MRONJ pathogenesis. Osteoclasts are osteoimmune cells with key roles in the development, homeostasis, and healing of bone. In the jaws, osteoclasts remove necrotic bone in the inflammatory milieu during dental disease or in extraction sockets and surgical sites. Given the close association of the jaws with the oral or sinus mucosa, osteoclast function is even more important in the oral environment, where pharmacologically mediated defective osteoclast function alters oral wound healing and plays a key role in macrophage malfunction (red arrows). Failure to remove necrotic bone and allow normal osteo-mucosal healing alters macrophage function, cytokine secretion, and polarization and propagates a pro-inflammatory, anti-resolving environment in a positive feedback mechanism that ultimately leads to MRONJ development, progression, and expansion.

Although macrophages’ role in MRONJ pathogenesis is undisputed, the extent of their involvement needs to be further investigated. Based on the results of current research, it is logical to assume that indirect and potentially direct effects of antiresorptives on macrophages modulate immune microenvironment at MRONJ developing areas, thus delaying wound healing and impairing angiogenesis or lymphangiogenesis. Understanding the complex role of macrophages in MRONJ pathophysiology will add new, valuable data on disease prevention and control.

## Figures and Tables

**Table 1:** Effect of antiresorptive medication on macrophages’ activity.

Bisphosphonates	Cellular Activity	Polarization	Protein Expression
THP1 cells	(↓) viability [33,52,53] (↓) migration [54] (↓) phagocytosis [54]	(↑) M1 (–) M2 [55] (↑) M1 (↓) M2 [16]	(↑) MMP9 (–) MMP2 [52]
RAW264.7 cells	(↓) viability [56] (↑) apoptosis [57] (↓) migration [58]		(↑) IL1 [59,60] (↑) (IL1b, IL6, TNFα,NO) [40]
BMDMs		(↑) M1 (↓) M2 [16]	(↑) (IL1b, IL6, TNFα) [61] (↑) IL1b [62] (↑) IL6 [63]
Mouse MRONJ models		(↑) M1 [16] (↑) M1\(↓) M2 [22] (↓) M2 [64]	(↑) (IL6, IL1b, caspase1) [60,62]
Human MRONJ tissue	(↓) differentiation [34]	(↑) M1 (↓) M2 [35] (↑) M1 [65]	(↑) IL6 (↓) IL10 [35]
Denosumab/ anti-RANKL			
THP1 cells	(–) viability [33]		
BMDMs	(–) viability [66] (–) number [67]		(–) IL10 (–)TGFb [67]
Mouse MRONJ models	(↓) wound healing [66,67] (–) differentiation [68,69] (↓) functionality [70]	(–) M1 (–) M2 [64] (↑) M1 (↑↑) * M2 [67]	
Human MRONJ tissue		(↑) M1 (↓) M2 [35]	(↑) IL6 (↓) IL10 [35]

(↑ upregulation, ↓ downregulation, – neutral); * when mAb was discontinued; BMDM, Bone marrow-derived macrophages.
